# Novel Genetic and Phenotypic Expansion in Ameliorated *PUF60*-Related Disorders

**DOI:** 10.3390/ijms25042053

**Published:** 2024-02-08

**Authors:** Emily Baum, Wenming Huang, Catherine Vincent-Delorme, Perrine Brunelle, Adam Antebi, Hormos Salimi Dafsari

**Affiliations:** 1 Department of Pediatrics, Faculty of Medicine and University Hospital Cologne, University of Cologne, 50937 Cologne, Germany; 2 Max-Planck-Institute for Biology of Ageing, 50931 Cologne, Germany; 3 Cologne Excellence Cluster on Cellular Stress Responses in Aging Associated Diseases (CECAD), 50931 Cologne, Germany; 4 Clinical Genetics Unit Guy Fontaine, University Hospital of Lille, F-59037 Lille, France; 5 Institut de Génétique Médicale, University of Lille, ULR7364 RADEME, CHU Lille, F-59000 Lille, France; 6 Department of Pediatric Neurology, Evelina’s Children Hospital, Guy’s & St. Thomas’ Hospital NHS Foundation Trust, London SE1 7EH, UK; 7 Randall Division of Cell and Molecular Biophysics, Muscle Signaling Section, King’s College London, London WC2R 2LS, UK; 8 Center for Rare Diseases, Faculty of Medicine and University Hospital Cologne, University of Cologne, 50937 Cologne, Germany

**Keywords:** *PUF60*, neurodevelopmental disorders, Verheij syndrome, immunological disorders

## Abstract

Heterozygous variants in the Poly(U) Binding Splicing Factor 60kDa gene (*PUF60*) have been associated with Verheij syndrome, which has the key features of coloboma, short stature, skeletal abnormalities, developmental delay, palatal abnormalities, and congenital heart and kidney defects. Here, we report five novel patients from unrelated families with *PUF60*-related disorders exhibiting novel genetic and clinical findings with three truncating variants, one splice-site variant with likely reduced protein expression, and one missense variant. Protein modeling of the patient’s missense variant in the PUF60 AlphaFold structure revealed a loss of polar bonds to the surrounding residues. Neurodevelopmental disorders were present in all patients, with variability in speech, motor, cognitive, social-emotional and behavioral features. Novel phenotypic expansions included movement disorders as well as immunological findings with recurrent respiratory, urinary and ear infections, atopic diseases, and skin abnormalities. We discuss the role of PUF60 in immunity with and without infection based on recent organismic and cellular studies. As our five patients showed less-severe phenotypes than classical Verheij syndrome, particularly with the absence of key features such as coloboma or palatal abnormalities, we propose a reclassification as *PUF60*-related neurodevelopmental disorders with multi-system involvement. These findings will aid in the genetic counseling of patients and families.

## 1. Introduction

Splicing is an essential step in eukaryotic messenger RNA (mRNA) processing that removes the intron regions of precursor transcripts to produce mature transcripts capable of encoding proteins [1]. This process is catalyzed by the spliceosome, a highly dynamic ribonucleoprotein complex composed of several splicing factors and small nuclear RNAs. The dysregulation of splicing impairs cellular function and is associated with cancers and aging [2,3]. Pathogenic variants in genes encoding core spliceosomal components have been shown to disrupt the splicing process and lead to various monogenic disorders with defective tissue development [4,5]. The Poly(U) Binding Splicing Factor 60 kDa gene (*PUF60*) encodes an essential splicing factor containing two central RNA recognition motifs (RRM1 and RRM2) and a C-terminal U2AF-homology motif (UHM, alternatively termed RRM3) [6]. PUF60 is involved at the early stage of spliceosome assembly by binding polyuridine (U) tracts and promoting the association of precursor transcripts with the U2 small nuclear ribonucleoprotein complex [7]. In vitro models have indicated the role of PUF60 in cellular migration, proliferation, and viability [8]. Furthermore, PUF60 overexpression was associated with different types of tumorigeneses [9,10].

Heterozygous loss-of-function variants in *PUF60* have been associated with Verheij syndrome (VRJS, OMIM#615583) since the first report of an 8.35 Mb deletion at the genetic location 8q24 with the key features of coloboma, congenital cardiac defects, neurodevelopmental delay, seizures, short stature, and skeletal anomalies [11]. Additional features from follow-up reports identified *PUF60* as the primary driver for this phenotype and included microcephaly, craniofacial dysmorphia, and genitourinary abnormalities consisting of congenital anomalies of the kidney and urinary tract [11,12]. To date, 73 cases have been reported in the literature. *PUF60* loss-of-function variants lead to weak 3′-splice site recognition, promoting aberrant splicing in which PUF60 deficiency dysregulates the alternative splicing of selected genes [6]. Animal models of PUF60 deficiency showed phenotypes similar to the human disorder, including decreased body size, growth retardation, and delayed development [13]. Additional phenotypes from a *Caenorhabditis elegans* model of its homolog *rnp-6* included immunodeficiency which was previously not associated with human *PUF60*-related disorders [14]. Here, we report five novel patients with *PUF60*-related disorders with a genotypic and phenotypic expansion of this ultra-rare genetic disease, including immune dysregulation and dermatological abnormalities. Our study establishes a much milder phenotypic spectrum that prompts us to propose a novel classification of *PUF60*-related disorders.

## 2. Results

### 2.1. Phenotype Expansion

**Figure 1:**
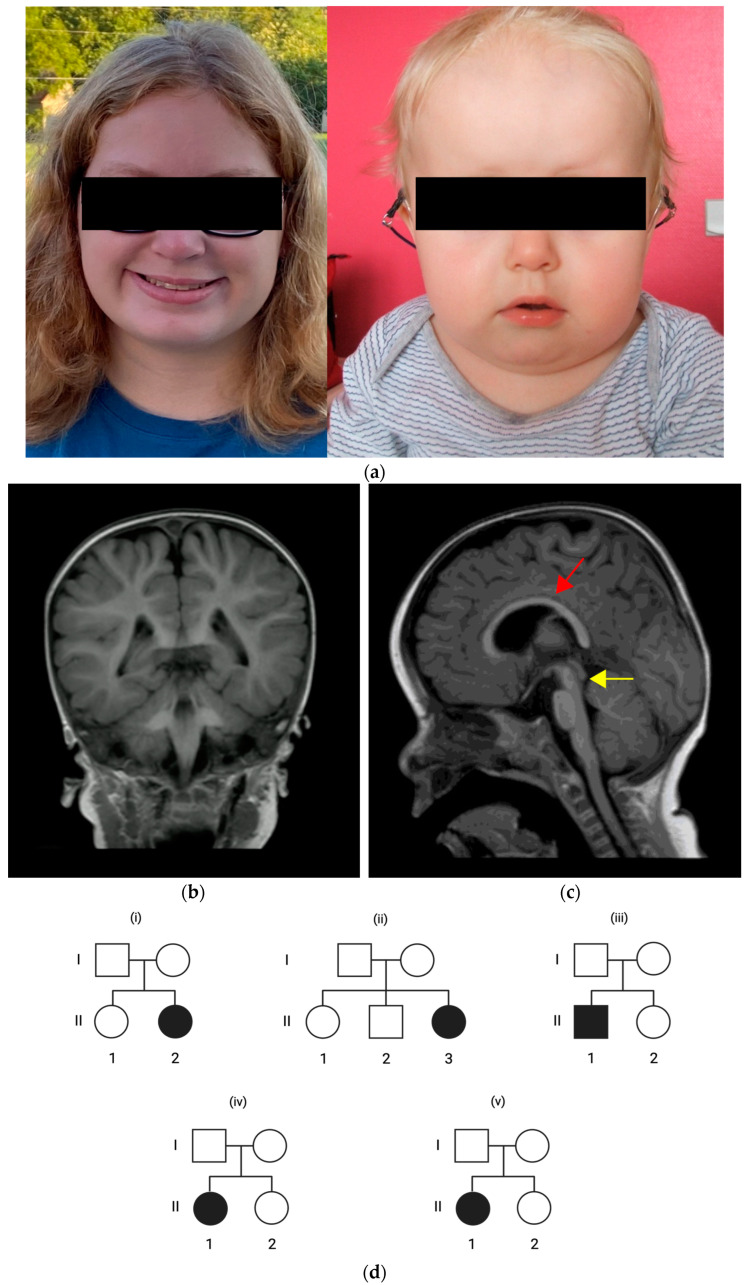
(**a**) Facial morphologies of patient 2 with full cheeks (left) and patient 3 with high forehead, wide depressed nasal bridge and full cheeks (right). (**b**,**c**) Axial brain magnet resonance imaging (MRI) in patient 3 at the age of 2 years with mesencephalon hypoplasia (yellow arrow) and corpus callosum dysgenesis (red arrow). (**d**) Pedigrees of patient 1 (i), patient 2 (ii), patient 3 (iii), patient 4 (iv), and patient 5 (v) indicating an inheritance pattern with healthy parents and de novo *PUF60* variants in the patients.

We identified five previously unreported patients from a multi-center study and follow-up online clinics. The pedigrees of the patients’ families are illustrated in Figure 1d. Table 1 summarizes the clinical findings in our patient cohort.

Patient 1 is a four-year-old female from non-consanguineous American parents without any notable pregnancy or perinatal history. She was first presented with speech neurodevelopmental delay with two-syllable words at 19 months and short sentences at 24 months, as well as unremarkable age-appropriate motor and cognitive development. Social, emotional, and behavioral developmental disorders include hand-waving stereotypies, aggressive behavior, and temper tantrums with self-injuring. She presented with pes planus and a generalized muscular hypotonia. The patient has a short stature despite adequate caloric intake, without any feeding problems or failure to thrive. The patient’s history included eight until ten infections per year, mostly urinary and ear infections. Tympanostomy and placement of tympanic tubes led to a slight reduction in the severity of ear infections. We observed four café-au-lait spots on the lower extremities. She presented without microcephaly, craniofacial dysmorphia, and ophthalmological, hearing, or palatal abnormalities. We identified no cardiac, respiratory, genitourinary, or gastrointestinal abnormalities on examination. There was no history of seizures. Electroencephalogram (EEG) recordings were unremarkable. Brain magnetic resonance imaging (MRI) did not reveal any brain malformations. A lumbar spine MRI showed the partial sacralization of L5, a thickened fatty filum terminale most prominent at L2–L3, and a tethered cord. Panel-based exome sequencing revealed a de novo heterozygous variant in *PUF60* (c.206del, (p.Lys69ArgfsTer16)).

Patient 2 is a 15-year-old female from non-consanguineous American parents without any notable pregnancy or perinatal history. She presented with generalized muscular hypotonia and an attention disorder. Milestones in speech, motor, and cognitive development were age-appropriate. On clinical presentation, we observed craniofacial dysmorphia, including full cheeks and almond-shaped eyes (Figure 1a), without palatal anomalies or microcephaly. Clinical examination revealed hyperelasticity of the skin, joint laxity, keratosis on the upper and lower extremities, acne, and two café-au-lait spots on the thigh. At the age of 15 years, she now reports muscle spasms in her extremities and an increasing loss of fine motor skills in her grip. The patient has one to six episodes of pneumonia per year. She has myopia and reports occasional blurred vision that coincides with viral infections. Her hearing is unremarkable. The patient showed no short stature, feeding problems, or failure to thrive. She reported a history of seasonal allergies, chronic gastritis, and insomnia. We observed no cardiac, respiratory, or genitourinary anomalies. We identified hemivertebrae, a 13th pair of ribs, and scoliosis, leading to two spinal surgeries in 2018 and 2020. She has a history of seizures with onset at two years old, although she is now seizure-free without anti-epileptic treatment. An EEG recording showed abnormal waves without epileptic discharges. A brain MRI was not performed. Panel-based exome sequencing revealed a de novo heterozygous variant in *PUF60* (c.1658A>G, (p.Asp553Gly)). 

Patient 3 is a three-year-old male from non-consanguineous French parents without any notable pregnancy or perinatal history. On the first clinical presentation, the patient had generalized muscular hypotonia and moderate bilateral hearing loss at the age of five months which prompted the use of hearing aids. The patient’s history indicated monthly episodes of moderate-to-severe laryngitis. We identified serous otitis media, middle ear hypoplasia, and bilateral external ear canal stenosis on examination. Tympanic tubes were placed at the age of two years. Audiometric re-evaluation indicated only mild hearing loss, and the use of hearing aids was stopped. There were no abnormalities in speech, motor, and cognitive developmental milestones; however, behavioral abnormalities included anxiety, stereotypies (i.e., repetitive movements), aggressive behavior, and temper tantrums with self-injuring. Furthermore, we observed an intention tremor upon neurological examination. The patient has a short stature with delayed bone age, and had no feeding problems or failure to thrive. The patient showed craniofacial dysmorphia, including a wide and depressed nasal bridge, a high forehead, and full cheeks (Figure 1a). He showed no microcephaly nor palatal, skin, or ocular abnormalities. We also identified no cardiac, respiratory, genitourinary, or gastrointestinal anomalies. The patient had no history of seizures. EEG recordings showed no remarkable findings. An MRI brain scan revealed corpus callosum dysgenesis and mesencephalon hypoplasia (Figure 1b,c). Panel-based exome sequencing revealed a de novo heterozygous variant in *PUF60* (c.636_640del, (p.Gln212HisfsTer7)).

Patient 4 is a 15-year-old female from non-consanguineous American parents without any notable pregnancy history. She was born preterm at 35 weeks without any notable cardiorespiratory abnormalities. The patient shows dysmorphic facial features, including almond-shaped eyes and plagiocephaly, without microcephaly or palatal abnormalities. The patient’s history indicated severe sleep apnea with daytime fatigue. She has a short stature and exhibited feeding problems during infancy without failure to thrive. She received growth hormone therapy at the age of four years. We identified obstipation as well as gluten and lactose intolerance. She showed a speech developmental delay, with two-syllable words at 18 months and short sentences at 24 months, and receives speech therapy and special education services. Formal non-verbal IQ testing revealed a learning disorder. She had adequate age-appropriate motor milestones. Her hearing was unremarkable, and an ophthalmological evaluation indicated myopia. On clinical examination at the age of 15 years, she presented with an out-toeing gait disorder, generalized muscular hypotonia, developmental coordination disorder, and attention deficit hyperactivity disorder (ADHD). We observed no cardiac or genitourinary abnormalities. The patient had no history of seizures. An EEG recording indicated sharp waves without discharges. A brain MRI did not show remarkable findings. Panel-based exome sequencing revealed a de novo heterozygous variant in *PUF60* (c.1123C>T, (p.Gln375Ter)).

Patient 5 is a 35-month-old female from non-consanguineous American parents without any notable pregnancy history. At term birth, she had an acute hypoxic bradycardic event with resuscitation and intensive care treatment. Cardiologic examinations revealed Tetralogy of Fallot and a right aortic branch, which prompted surgical procedures. She has plagiobrachycephaly without microcephaly, and received helmet therapy. She shows dysmorphic facial features, including a prominent forehead, a square face, full cheeks, anteverted nares, a thin upper lip, and a short nose with a broad nasal root and tip. We identified no palatal abnormalities. She showed mild generalized hypotonia, gross motor development delay with crawling at 19 months and independent walking at two years, speech delay with the last milestone of two-word phrases at 35 months, and a behavioral disorder with stereotypic eye-rolling. The patient received speech and physical therapy. Immunological features included eczema and macular skin rashes, allergy to eggs, asthma, inspiratory stridor, recurrent pneumonia, and otitis media. Tympanostomy and the placement of tympanic tubes occurred at the age of 18 months. Her hearing was unremarkable. The patient has a short stature. The patient had feeding difficulties with gastroesophageal reflux disease (GERD) and feeding via a percutaneous endoscopic gastrostomy (PEG) tube until the age of 15 months. We identified bilateral congenital hip dysplasia, a Klippel–Feil anomaly, a Sprengel deformity, a cervical spine fusion at C2/3, and hypoplastic C1 vertebrae. We identified a delayed bone age with more than two standard deviations below chronological age. The patient has joint laxity, a tethered cord, and an acquired bilateral ankle pronation. We observed clitoromegaly without any other genitourinary abnormalities. An EEG recording was unremarkable. Her brain MRI scan revealed corpus callosum dysgenesis and mild white-matter volume loss. Panel-based exome sequencing revealed a de novo heterozygous variant in *PUF60* (c.510+1G>T, (p.?)).

### 2.2. Molecular Genetic Spectrum and Protein Modeling

**Figure 2:**
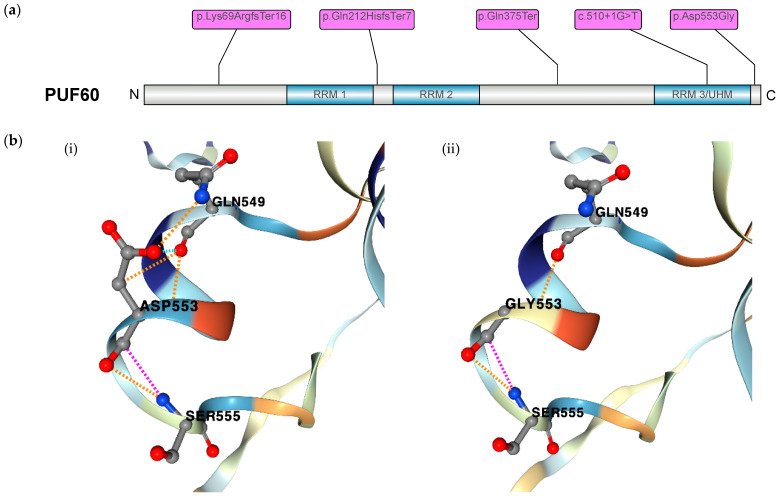
(**a**) Schematic of PUF60 protein motif structure with the position of novel variants of the five patients indicated. (**b**) Protein modeling of the missense variant in (p.Asp553Gly) with (i) view of wildtype residue Asp553 and (ii) loss of polar bonds (illustrated in orange) between mutated residue Gly553 and Gln549.

We identified a molecular genetic spectrum with two frameshift-truncating, one truncating, one splice-site, and one missense variant in *PUF60* (Figure 2a). Co-segregation for the disorder within the family was performed via dideoxy sequencing with standard human genetic consultations in order to examine for de novo variants in all five cases. We have added the *PUF60* variants to the ClinVar database [15].

The frameshift-truncating, truncating, and splice-site variants in *PUF60* are predicted to lead to reduced protein expression. The variant (p.Lys69ArgfsTer16) is located at the N-terminal region before the RRM1 domain. The variant (p.Gln212HisfsTer7) is located between the domains RRM1 and RRM2. The truncating variant (p.Gln375Ter) is located between the domains RRM2 and RRM3/UHM. The splice-site variant is located in an exon that encodes for amino acids in the RRM3/UHM domain. The missense variant is located at the C terminus. Inspection of the AlphaFold2-predicted structural model of PUF60 showed a pathogenic effect of the missense mutation in patient 2 on the protein level. Moreover, (p.Asp553Gly) was predicted to incur a destabilizing effect (ΔΔGStability of −0.59 kcal/mol) with a loss of hydrogen bonds to Gln549 in mutant Gly553 when compared to wildtype Asp553 (Figure 2b). 

## 3. Discussion

In this study, we report five novel patients with pathogenic *PUF60* variants with deep phenotyping and previously unreported features that expand the entity of *PUF60*-related disorders. We propose a reclassification of *PUF60*-associated disorders since the clinical features of our patients are milder than the classical Verheij syndrome phenotypes. 

Since its original report in 2009, Verheij syndrome has mainly been characterized by neurodevelopmental delay, short stature, craniofacial dysmorphia, and skeletal abnormalities [11]. Several case series have identified *PUF60* as the monogenic cause of Verheij syndrome and mainly reiterated the phenotypic findings [16]. The comprehensive genotype–phenotype spectrum in this disorder remains elusive due to limited reports. In our cohort of five novel patients, we observed two frameshift-truncating, one truncating, one splice-site, and one missense variant in *PUF60* (Figure 2a). The truncating variants are predicted to lead to a decrease in protein expression, as illustrated in previous reports [6]. We also examined the pathogenic effect of the missense variant on protein stability. The inspection of the AlphaFold2-predicted structural model of PUF60 indicated a loss of polar bonds between mutated residue Gly553 and surrounding residue Gln549, which likely leads to loss of function (Figure 2b). We analyzed the phenotypic spectrum in regard to either the location of mutations or the altering effect (truncation vs. missense mutation) and did not observe a clear genotype–phenotype correlation. The pleiotropy in phenotypes was previously reported in two cohorts with phenotypic frequencies that are comparable to our cohort [16,17]. 

Generalized muscular hypotonia was present in all our patients, in one case leading to feeding problems due to prominent orofacial hypotonia and prompting PEG tube feeding. Neurodevelopmental disorders were examined in all four domains: (i) speech, (ii) motor, (iii) intellectual/cognitive, and (iv) social, emotional, and behavioral development. Of note, all but one patient manifested a speech developmental delay, while only two patients showed motor developmental delays, and one patient had an intellectual disability. We identified disorders in social, emotional, and behavioral development, including aggressive behavior with self-injuring in patients 1 and 3; attention deficits in patients 2 and 4; and stereotypies in patients 1, 3, and 5. Previous studies have reported only a few cases with temper tantrums and self-injuring behavior, and here, we observed a much higher frequency in our cohort [17,18]. We propose that, if considering all domains of neurodevelopmental disorders, this phenotype is a key feature in diagnosing even milder *PUF60*-related disorders, and we consider it the singular core feature in the re-classification of the spectrum of *PUF60*-related disorders (Table 2).

Brain malformations are present in a number of *PUF60* variants in the literature [11,12,16,17,18,19,20,21,22,23]. Only patient 3 presented with brain malformations, including corpus callosum dysgenesis and mesencephalon hypoplasia (Figure 2b). The absence of brain malformations in three out of four patients from our cohort who had a brain MRI examination may show that brain malformations are indeed a less frequent feature. We also report an age-dependent onset of movement disorders in the phenotype expansion, as patient 2 reported muscle spasms in her extremities and an increasing loss of fine motor skills in her grip at the age of 15 years. Of note, patient 3 also showed an intention tremor upon neurological examination, but there was no corresponding cerebellar abnormality in the brain MRI. We argue that any diagnostic work-up at the initial presentation of *PUF60*-related disorders should prompt a brain MRI examination.

Seizures were one of the key features in the first description of classical Verheij syndrome and other reports [11,12,16,17,18,23,24,25,26]. Only one patient in our cohort presented with a history of seizures until the age of two years, and remained seizure-free without any medication until the present age of 15 years. Two patients had abnormal EEG findings, whereas one of these patients had no clinical history of seizures. Hence, any initial diagnostic evaluation of *PUF60*-related disorders should indeed include EEG findings.

We present a phenotypic expansion with novel immunological findings in our cohort. Patient 1 had recurrent infections from infancy, with a peak at two years of age that included eight to ten ear infections per year. Patient 2 had six flu-like infections per year as well as acne and keratosis. Patient 3 had eight bouts of laryngitis per year with serous otitis media. Patient 4 had up to six bouts of pneumonia per year during early childhood. Patient 5 had more than two bouts of pneumonia per year as well as eczema, macular skin rashes, asthma, allergy to eggs, and inspiratory stridor. Several patients had tympanostomy with the placement of tympanic tubes, which led to a general improvement in the severity and frequency of ear infections, even leading to a change from a moderate to a mild hearing disability in one patient. As the frequency of hearing loss was much higher in previous cohorts with *PUF60*-related disorders, we suggest an early audiometric evaluation at initial diagnostic assessments and a constant re-evaluation of ear infections as well as potential surgical procedures to alleviate the course of chronic otitis media [27]. Notably, immunological phenotypes might have been overlooked in the evaluation of *PUF60*-related disorders, as only one case with recurrent pneumonia and upper respiratory tract infections had been reported before [12]. We analyzed the immunological phenotypes according to guidelines for screening warning signs in patients with primary immunodeficiency [28,29]. Patients 1 and 3 had ≥4 ear infections in one year. Patient 4 had ≥2 bouts of pneumonia in one year. Skin abnormalities including eczema, skin rashes, and keratosis were found in patients 2 and 5, and they are increasingly being recognized as warning signs in patients with primary immunodeficiency [30,31]. Allergic manifestations in patients with primary immunodeficiency include food allergies and asthma, which were both found in patient 5 [32]. Additionally, short stature has been recognized as a rather frequent feature in a large collective of patients with primary immunodeficiency, and four out of five patients in our cohort showed short stature [33,34]. Recent guidelines in the field of primary immunodeficiency have recommended rigorous screening for primary immunodeficiency if two or more warning signs are present in patients with recurrent infections [31]. As each of our five patients showed two or more warning signs, we propose to include immunological assessments (complete blood count, immunoglobulins, T/B/NK subsets with FACS) at initial presentation and/or diagnosis of a *PUF60*-related disorder to assess for any signs of immunodeficiency, especially with a patient history of recurrent infections [35,36,37,38].

PUF60 knockdown in HeLa cells without infections induced the pro-inflammatory cytokines IL-8, IL-6, CXCL2, and IL-1α [14]. This may suggest that patients with PUF60 deficiency have increased levels of pro-inflammatory cytokines in the absence of an infection that potentially correlates with exaggerated immunological responses during asthma, eczema, or atopic diseases. At six hours after an *S. aureus* infection, PUF60 expression was significantly reduced in wildtype HeLa cells. HeLa cells with a mutation in PUF60:(p.Gly300Asp) were infected with *S. aureus* and also displayed a significantly compromised induction of pro-inflammatory cytokines IL-8, IL-6, CXCL2, and IL-1α [14]. *Caenorhabditis elegans* models with a (p.Gly281Asp) missense mutation in its homolog RNP-6 revealed decreased life spans on infection with *Staphylococcus aureus*, *Pseudomonas aeruginosa*, and *Enterococcus faecalis* [14]. Analyses of qRT-PCR experiments revealed the compromised induction of innate immunity genes *nlp-30*, *nlp-34*, *spp-1*, and *ilys-2* upon *S. aureus* infection [14]. This may suggest that patients with PUF60 deficiency have a dysregulated immunological response during bacterial infections of the respiratory tract, ears, or urinary tract, as observed in our cohort. 

Patients 1 and 2 had café-au-lait spots. Patients 2 and 5 had joint laxity. Additional dermatological features included hyperelasticity of the skin and keratosis on the upper and lower extremities in patient 2. We discuss that the syndromic appearance of recurrent infections and dermatological abnormalities akin to hypermobile Ehlers–Danlos syndrome may suggest a deficient immunological response in the skin, as these findings have previously been reported with a link to immunodeficiency in other disease entities [39,40].

Short stature was reported as a key feature of classical Verheij syndrome. However, we observed a normal stature in patient 2, potentially changing short stature from a core feature to a supportive feature in the diagnosis of *PUF60*-related disorders. Patient 4 was treated with growth hormones, marking the first successful treatment reported in the literature to our knowledge. One patient was previously reported to have growth hormone deficiency upon stimulation testing, as well as lower IGF1 and IGFBP3 levels, but did not receive growth hormone therapy [41]. Patient 4 also presented with fatigue after growth hormone treatment. We discuss that this presentation is most likely due to the sleep apnea in this patient with generalized muscular hypotonia and does neither indicate a side effect of the treatment nor a phenotypic expansion in *PUF60*-related disorders [42]. 

Another key feature of classical Verheij syndrome is iris coloboma. Previous reports had already reported a lower frequency of coloboma [18]. Our cohort reiterates this finding with an absence of coloboma and instead reports the presence of several other ophthalmological phenotypes, such as transient blurred vision during viral infections. None of our five patients had any microcephaly, genitourinary, or cardiac defects. Syndromic microcephaly may present concomitantly with neurodevelopmental disorders [43]. The frequency of cardiac or genitourinary abnormalities is estimated at around 5% in the general public, and is associated with a number of chromosomal deletions or monogenic disorders [44,45]. In a recent report of ten patients, two patients had genitourinary abnormalities and four patients had cardiac defects [18]. The noted absence of cardiac or genitourinary abnormalities in our cohort again illustrates that the core *PUF60*-related phenotype does not necessarily consist of cardiac or genitourinary phenotypes. This again supports the much milder phenotype of pathogenic *PUF60* variants in our patients that does not include the full picture of classical Verheij syndrome.

Hence, we propose to reclassify this entity as *PUF60*-related neurodevelopmental disorders with additional multi-system involvement and delineate mild *PUF60*-related neurodevelopmental disorders from classical Verheij syndrome.

## 4. Materials and Methods

### 4.1. Patient Recruitment

The study was approved by local institutional review boards. All patients and/or their legal guardians gave informed consent, including consent to the anonymous publication of their clinical information [46]. Where photographs are shown, we obtained written informed consent for the publication of photographs from all participants and/or their legal guardians.

### 4.2. Molecular Genetic Investigations

Genetic variants were identified via gene panel investigations based on massively parallel sequencing [47,48]. All variants were analyzed in detail using in silico pathogenicity prediction scores via multiple lines of bioinformatic tools [49,50]. All variants were absent from the healthy population database (gnomAD version 3.1.2) [51]. All patient variants were classified as pathogenic according to the standards and guidelines of the American College of Medical Genetics and Genomics for the interpretation of sequence variants [52].

The linear protein model was visualized with the IBS illustrator tool [53]. We conducted protein modeling on the missense variant in patient 3 in wildtype and mutant form with DynaMut2 to assess pathogenicity in patient mutations. The mutation Asp553Gly was modeled in AlphaFold structure AF-Q9UHX1-F1. We additionally examined the computed destabilizing effect that was predicted by Dynamut2, as indicated with ΔΔGStability (kcal/mol) [54].

## 5. Conclusions

In conclusion, we present five novel patients with new phenotypes and previously unreported genetic variants, further delineating the mutational spectrum and phenotypic variety of *PUF60* variants. The phenotypic expansion includes immunological phenotypes that should be considered in the diagnostic evaluation of *PUF60*-related disorders. We propose to re-classify the disease entity as PUF60-related neurodevelopmental disorders with additional multi-system involvement.

## Figures and Tables

**Table 1:** Overview of core features of patients from this study. Abbreviations: f = female, m = male, het. = heterozygous, y = years; m = months; + = present; − = not present; n/a = information not available.

Features	Patient 1	Patient 2	Patient 3	Patient 4	Patient 5
***PUF60* variant** (NM_078480.3, NP_510965.1)	de novo het., c.206del, (p.Lys69ArgfsTer16)	de novo het., c.1658A>G, (p.Asp553Gly)	de novo het., c.636_640del, (p.Gln212HisfsTer7)	de novo het., c.1123C>T, (p.Gln375Ter)	de novo het., c.510+1G>T, (p.?)
**Sex**	f	f	m	f	f
**Age last reported**	4 y	15 y 6 m	3 y	15 y	35 m
**Age at presentation**	3 y	2 y	5 m	3 y	at birth
**Initial symptom**	Neurodevelopmental delay	Seizures	Bilateral hearing Loss	Neurodevelopmental delay	Tetralogy of Fallot
**Neurological manifestations**					
Muscular hypotonia	+	+	+	+	+
Speech developmental disorder	+	+	−	+	+
Motor developmental disorder	−	−	−	+	+
Intellectual developmental disorder	−	−	−	+	−
Social, emotional or behavioral developmental disorder	+	+	+	+	+
Brain malformations	−	n/a	+	−	+
Tremor	−	−	+	−	−
Seizures	−	+	−	−	−
**Multi-system involvement**					
Recurrent infections	+	+	+	−	+
Short stature	+	−	+	+	+
Skin abnormalities	+	+	−	+	+
Gastrointestinal abnormalities	−	+	−	+	+
Skeletal abnormalities	+	+	−	−	+
Joint laxity	−	+	−	−	+
Genitourinary abnormalities	−	−	−	−	+
Cardiac abnormalities	−	−	−	−	+
**Facial manifestations**					
Craniofacial dysmorphia	−	+	+	+	+
Microcephaly	−	−	−	−	−
Palatal abnormalities	−	−	−	−	−
Coloboma	−	−	−	−	−
Other ophthalmological abnormalities	−	+	−	−	+
Hearing loss	−	−	+	−	−

**Table 2:** Overview of the proposed re-classification of *PUF60*-related disorders with core and supportive features, shifting the paradigm from the previously annotated classical Verheij syndrome as a full phenotypic picture towards milder *PUF60*-related disorders with delayed speech, motor, intellectual, and/or social, emotional and behavioral development.

Core Features	
	Pathogenic heterozygous *PUF60* variant
	(History of) neurodevelopmental disorder in speech, motor, intellectual, and/or social, emotional and behavioral development
**Supportive features indicating multi-system involvement**	
Craniofacial abnormalities	Microcephaly
	Palatal abnormalities
	Craniofacial dysmorphia
	Coloboma and/or other ophthalmological abnormalities
	Hearing loss
Neurological manifestations	Brain malformations
	Seizures
	Fatigue
	Movement disorders
	Muscular hypotonia
Skeletal abnormalities	Short stature
	Joint laxity
	Vertebral fusion, Sprengel deformity and/or scoliosis
Organ abnormalities	Congenital heart defects
	Gastrointestinal abnormalities
	Congenital anomalies of kidney and urinary tract
Immunological features	Recurrent infections
	Skin abnormalities
	Atopic diseases

## Data Availability

Anonymized data are available upon request from the corresponding author.
